# Consent for genomic sequencing: a conversation, not just a form

**DOI:** 10.1038/s41431-025-01805-0

**Published:** 2025-03-03

**Authors:** Danya F. Vears

**Affiliations:** 1 School of Medicine, Deakin University, Geelong, VIC Australia; 2 Murdoch Children’s Research Institute, Parkville, VIC Australia; 3 Department of Paediatrics, University of Melbourne, Parkville, VIC Australia; 4 Centre for Biomedical Ethics and Law, KU Leuven, Leuven, Belgium

**Keywords:** Diagnosis, Patient education

How to obtain truly informed consent for genomic sequencing in clinical practice is a long-standing topic of debate in the field. Although some research (including interviews with health professionals and analysis of consent forms) has already been conducted in this space, these studies can often only give a vague indication of the nature of the information that patients or families might be provided. They are limited in that they assume that health professionals use the consent form as a template to guide the discussion with the patient when, in reality, very little research has been conducted that examines exactly how, if at all, consent forms are utilised in these consultations. The study conducted by Ellard et al. [1] goes a step further than most to explore what happens during a consent interaction when patients are offered diagnostic genomic sequencing within a public health service. Their team audio-recorded consent conversations between healthcare professionals and parents of paediatric patients offered WGS through the Genomic Medicine Service across seven regionally diverse NHS Trusts in the United Kingdom. However, it also raises further questions, including how to make a consent interaction into a legitimate conversation and what information is important to convey during this conversation.

Previous research in this space, such as analyses of consent forms available online [2, 3], has suggested that information presented to patients or families varies considerably between genetic services. Interviews with health professionals have also highlighted that they question whether fully informed consent is possible at all given the complex nature of genomic sequencing and the potential for identifying incidental findings [4]. Moreover, there has been some suggestion that health professionals might view consent forms as a way of protecting themselves from liability rather than as a way of obtaining truly informed consent [4].

These findings, among others, led my colleagues and I to propose that, rather than ensuring patients and their families have all of the information, health professionals should focus more on the information that is relevant to the patient/family in order to make an appropriately informed decision [5]. To achieve this, health professionals need to elicit the values and preferences of the patient/family and then target the information provided and the broader discussion to meet these needs.

Ellard and colleagues quantified the average proportion of words spoken by health professionals versus parents [1]. While the study team use the term ‘consent conversation’ to refer to this interaction, which would describe the ideal nature of the interaction, this seems generous given that the average proportion of words spoken by the parents was only 11%. Likewise, they noted that several of the health professionals did not prompt parents to ask questions or clarify their understanding in a meaningful way. As mentioned above, a key component of the ability to target the information to the family’s needs is the eliciting of the values of the patient/family. While we do not know the extent to which the families were known to the health professionals prior to the consent appointment, certainly in these recorded discussions it does not appear that the health professionals were tailoring the information based on the needs or values of the families.

Ellard et al. noted that most of the health professionals they observed covered concepts such as the possibilities of an uncertain result (25/26), or a health-related incidental finding (25/26), and not receiving a diagnosis (24/26) [1]. However, less than half addressed the concept that data may be reanalysed in the future (12/26). Why is that important? Well, genomic sequencing for diagnostic purposes is rapidly becoming a first line test in many areas of medicine for conditions suspected to have a genetic basis. Although a large proportion of patients do not receive a diagnosis the first time around, reanalysis of undiagnosed cases approximately 2 years later has shown increases in yield around 10% [6]. As such, professional bodies are beginning to advocate for reanalysis to become routine practice [7]. Currently, most genetic services are already swamped with requests for diagnostic GS and do not have capacity to reanalyse routinely. However, several groups around the world are developing automated pipelines in order to reduce the workload for laboratory scientists and the overall costs of reanalysis, making routine reanalysis a real possibility.

The proposal of automated reanalysis often raises questions within genetic services regarding exactly what testing patients (or their families) have given consent to. Research conducted by my team and some of our collaborators has shown that both health professionals and laboratory scientists hold mixed views on whether their patients have given adequate consent for reanalysis to occur (unpublished data). These health professionals flag consent as a concern, despite the fact that the consent forms they use do mention the possibility of future retesting [8]. Previous analyses of consent forms available online suggested that varying information was being provided to patients and their families about the potential for reanalysis to occur and the processes around how this might be triggered [2].

Going back to their findings, Ellard and colleagues suggest that the omission of certain points from the consent conversation might suggest a move towards a model of appropriately, rather than fully, informed consent [1]. However, our proposal for appropriately informing patients/families stipulates that there should be a ‘minimum list’ of information points to be covered and that any additional information should be targeted according to patient/family needs and values [5]. This ‘tiered’ approach has also been proposed by others [9]. Given the ever-increasing likelihood that reanalysis of data will occur in the not-too-distant future, one might suggest that information about the fact that their data may be relooked at down the track should be on the ‘minimum list’ to be covered.

Ellard et al. propose that genetic associates are, both from a training and an affordability perspective, well placed to have the consent conversation with patients and families. While I believe this to be true, we need more evidence that they are doing so in a way that achieves appropriately informed consent. For example, given that genetic associates and pre-registration genetic counsellors spent longer on the consent process, it would be interesting to know if parents also spoke more during these consultations. A conversational analysis would also be helpful to explore the ways in which these health professionals with counselling training elicit questions and gauge understanding from parents in this context. It would also be interesting to record the consent conversations visually to see how the form is actually used as a tool during the discussion, if at all. Having this information could help us better design forms to suit the way(s) in which they are being used.

While obtaining consent is a necessary component of the testing process, it is important to remember what the goals of doing so are: to respect and promote autonomy; to enhance wellbeing; and to maintain trust within the health professional/patient relationship, as well as in the healthcare system more broadly [5]. Ensuring that consent is a conversation, not just a form, is essential to achieve these goals.

## References

[1] Ellard H, Alfardus H, Sanderson S, Lewis C. What does a consent conversation for whole genome sequencing look like in the NHS Genomic Medicine Service? An observational study. Eur J Hum Genet. 2024. 10.1038/s41431-024-01749-x. 10.1038/s41431-024-01749-x PMC11985900 39592828

[2] Vears DF, Niemiec E, Howard HC, Borry P. Analysis of VUS reporting, variant reinterpretation and recontact policies in clinical genomic sequencing consent forms. Eur J Hum Genet. 2018;26:1743–51. 30143804 10.1038/s41431-018-0239-7 PMC6244391

[3] Vears DF, Niemiec E, Howard HC, Borry P. How do consent forms for diagnostic high-throughput sequencing address unsolicited and secondary findings? A content analysis. Clin Genet. 2018;94:321–9. 29888485 10.1111/cge.13391

[4] Vears DF, Borry P, Savulescu J, Koplin JJ. Old challenges or new issues? Genetic Health Professionals’ experiences obtaining informed consent in diagnostic genomic sequencing. AJOB Empir Bioeth. 2021;12:12–23. 33017265 10.1080/23294515.2020.1823906 PMC8120994

[5] Koplin JJ, Gyngell C, Savulescu J, Vears DF. Moving from ‘fully’ to ‘appropriately’ informed consent in genomics: the PROMICE framework. Bioethics. 2022;36:655–65. 35390218 10.1111/bioe.13027 PMC9321597

[6] Dai P, Honda A, Ewans L, McGaughran J, Burnett L, Law M, et al. Recommendations for next generation sequencing data reanalysis of unsolved cases with suspected Mendelian disorders: a systematic review and meta-analysis. Genet Med 2022;24:1618–29. 35550369 10.1016/j.gim.2022.04.021

[7] Deignan JL, Chung WK, Kearney HM, Monaghan KG, Rehder CW, Chao EC. Points to consider in the reevaluation and reanalysis of genomic test results: a statement of the American College of Medical Genetics and Genomics (ACMG). Genet Med 2019;21:1267–70. 31015575 10.1038/s41436-019-0478-1 PMC6559819

[8] Best S, Fehlberg Z, Richards C, Quinn MCJ, Lunke S, Spurdle AB, et al. Reanalysis of genomic data in rare disease: current practice and attitudes among Australian clinical and laboratory genetics services. Eur J Hum Genet. 2024;32:1428–35. 38796577 10.1038/s41431-024-01633-8 PMC11576731

[9] Bunnik EM, Janssens AC, Schermer MH. A tiered-layered-staged model for informed consent in personal genome testing. Eur J Hum Genet. 2013;21:596–601. 23169494 10.1038/ejhg.2012.237 PMC3658183

